# Technologies for Fertilizers and Management Strategies of N-Fertilization in Coffee Cropping Systems to Reduce Ammonia Losses by Volatilization

**DOI:** 10.3390/plants11233323

**Published:** 2022-12-01

**Authors:** Tainah Freitas, Lucas Bartelega, César Santos, Mateus Portes Dutra, Leonardo Fernandes Sarkis, Rubens José Guimarães, Anderson William Dominghetti, Pauliana Cristina Zito, Tales Jesus Fernandes, Douglas Guelfi

**Affiliations:** 1 Department of Agriculture, Federal University of Lavras, Lavras 37203-202, Brazil; 2 Department of Soil Science, Federal University of Lavras, Lavras 37203-202, Brazil; 3 Department of Agriculture, Federal Institute of Espírito Santo, Santa Maria de Jetibá 29645-000, Brazil; 4 Department of Statistics, Federal University of Lavras, Lavras 37203-202, Brazil

**Keywords:** N-fertilizers, NH_3_ emission, urease inhibitors, slow- and controlled-release N-fertilizers, *Coffea arabica*, sustainable agriculture

## Abstract

The aim of this study was to quantify NH_3_-N losses from conventional, stabilized, slow-release, and controlled-release N fertilizers in a coffee field. The N fertilizers analyzed were prilled urea, prilled urea dissolved in water, ammonium sulfate (AS), ammonium nitrate (AN), urea + Cu + B, urea + adhesive + CaCO_3_, and urea + NBPT (all with three split applications), as well as blended N fertilizer, urea + elastic resin, urea-formaldehyde, and urea + polyurethane (all applied only once). NH_3_-N losses (mean of two crop seasons) were statistically higher for urea + adhesive + CaCO_3_ (27.9% of applied N) in comparison with the other treatments. Loss from prilled urea (23.7%) was less than from urea + adhesive + CaCO_3_. Losses from urea + NBPT (14.5%) and urea + Cu + B (13.5%) were similar and lower than those from prilled urea. Urea dissolved in water (4.2%) had even lower losses than those treatments, and the lowest losses were observed for AS (0.6%) and AN (0.5%). For the single application fertilizers, higher losses occurred for urea + elastic resin (5.8%), blended N fertilizer (5.5%), and urea + polyurethane (5.2%); and urea-formaldehyde had a lower loss (0.5%). Except for urea + adhesive + CaCO_3_, all N-fertilizer technologies reduced NH_3_-N losses compared to prilled urea.

## 1. Introduction

Brazil is the largest coffee (*Coffea arabica* L.) producer and exporter worldwide, and the constant search for better beverage quality and sustainability is essential in different coffee production systems. The application of nitrogen (N) fertilizers is imperative to achieve an adequate yield of this high-value crop. Nitrogen is the nutrient most extracted by the coffee plant and the nutrient of second greatest export by coffee beans [1,2].

Some studies using ^15^N have shown that coffee plants take up less than 25% of the N fertilizer when applied as conventional urea [3,4]. The dynamic transformation of N forms in the soil and the varying pathways of N losses in coffee growing areas result in low N fertilizer use efficiency (NUE) [4]. Ammonia (NH_3_-N) volatilization is the primary N loss in coffee production areas in Brazil [2,5,6], particularly when conventional urea is applied on the soil surface with plant residues and without fertilizer incorporation [7]. This is a common practice in systems of perennial crops such as coffee.

In 2017, the amount of N fertilizers used in the world was estimated at 150 Tg N per year [8], and may reach 260 Tg N per year in 2050 [9]. About 50% of global N fertilizer production is represented by urea [10,11]. The NH_3_-N losses from urea can be intensified when specific soil properties are combined with climatic conditions favorable to this loss. Such properties and conditions include the application of urea on moist soil followed by an absence of rainfall, increased soil and air temperature, increased N doses, application of N on soils with low cation exchange capacity, and alkaline soil pH [12,13]. NH_3_-N losses can exceed 50% of the applied dose, considering N applications in multiple crops [14,15]. The equivalent of one in three applications of N fertilizer is lost by volatilization in coffee production systems using conventional urea as an N source [2,5,16].

NH_3_-N losses not only reduce NUE and cause agronomic damage, but also lead to environmental problems. These problems include air pollution, due to the acidifying nature of NH_3_ [17], and greenhouse gas emissions to the atmosphere. NH_3_ gas is an indirect source of nitrous oxide (N_2_O), which has a global warming potential 310 times greater than carbon dioxide (CO_2_) [18]. It is estimated that 1.4% of the total volatilized N is converted to or lost as N_2_O [19].

The 4R principles (right nutrient source, right rate, right time, and right place) guide various management practices to minimize nutrient losses and the C footprint and increase N retention in the soil [20]. The development and proper use of enhanced-efficiency fertilizers (EEFs) may reduce these N losses [20,21,22]. Technological development of fertilizers is currently one of the strategies most investigated for improving NUE [23,24,25].

The fertilizer technology market had a compound annual growth rate estimated at 12% from 2014 to 2020 [26]. In addition, some European countries, including Germany and the Netherlands, have already adopted measures banning the application of conventional urea without incorporation and encouraging the use of some technologically enhanced fertilizer.

Enhanced efficiency fertilizers are in four main categories: stabilized fertilizers, slow-release fertilizers, controlled-release fertilizers, and their blends [27]. Stabilized fertilizers can inhibit some stages of N transformation in the soil through additives such as urease or nitrification inhibitors (e.g., N-(n-butyl) thiophosphoric triamide—NBPT) [28,29]. Some chemical compounds, such as boric acid, and metallic ions, such as copper (Cu), may also function as urease inhibitors when used in adequate concentrations [28,30].

The slow-release or chemically modified fertilizers are products of condensation of urea with aldehydes (e.g., formaldehyde and acetaldehyde). Controlled-release fertilizers are those with coatings that control the release of nutrients by diffusion or by a physical barrier (e.g., sulfur, wax, and polymer) [24,31]. In addition to these technologies, combining enhanced efficiency fertilizers and conventional N sources gives rise to another category of fertilizers, what are known as blends. N fertilizer blends are produced from the physical mixture of different fertilizer technologies with conventional N sources (stabilized, slow-release, or controlled-release fertilizers). Combining these N sources has many advantages, including a reduction in production costs compared to separate application of slow- or controlled-release fertilizers, the optimization of dynamics between nutrient release and plant nutrient uptake, and a reduction in NH_3_-N losses compared to conventional urea [32,33].

In addition to the different technologies available on the market, diverse N fertilization application strategies can be used, such as the mechanical incorporation of fertilizers into the soil. However, incorporation cannot be used in some cropping systems. In coffee fields, for example, mechanical incorporation of fertilizers may damage the root system, whose greatest activity occurs in the first 0.30 m of the soil [34]. Thus, urea dissolved in water, applied via a jet directed to the soil, can be a promising alternative, since urea would be automatically incorporated.

In this study, we tested the hypothesis that enhanced efficiency N fertilizers and other fertilizers, such as ammonium nitrate and sulfate and prilled urea diluted in water, are options more suitable than conventional urea to reduce NH_3_-N losses in coffee production systems. We chose the main technologies available on the market to perform this study. Thus, the objective of this study was to quantify NH_3_-N losses by volatilization from conventional, stabilized, slow-release, and controlled-release N fertilizers, as well as from a fertilizer blend, that were applied for two crop seasons on a coffee growing area in the production stage.

## 2. Results

### 2.1. Weather Conditions

The accumulated precipitation in the fertilization period of the 1st year was: 337 mm, 289 mm, and 69 mm at the 1st, 2nd, and 3rd split fertilizations, respectively, totaling 694 mm. In the first seven days after each fertilizer application, these rainfall values corresponded to 78 mm (23%), 171 mm (59%), and 19 mm (27%) (Figure S1). In the 2nd year, the rainfall accumulated during the fertilization period was: 153 mm, 124 mm, and 178 mm at the 1st, 2nd, and 3rd split fertilizations, respectively, totaling 455 mm. In the first seven days after each fertilizer application, these rainfall values corresponded to 102 mm (67%), 64 mm (52%), and 40 mm (22%) (Figure S3). The mean annual rainfall over the two years of assessment was 1243.3 mm.

The relative air humidity was higher than the critical relative humidity of urea (75%) for most of the period after fertilization in the two crop seasons. The mean temperatures in the same period were 21.2 and 22.6 °C in the first and second crop seasons, respectively. The minimum temperatures were 19 and 18 °C, and the maximum temperatures were 28 and 25 °C. Between the years 2015 and 2017, January had the highest mean temperature (30 °C), and June had the lowest mean temperature of (14 °C). The potential evapotranspiration (ETP) was around 899 to 873 mm per year [35].

### 2.2. Daily and Accumulated N-NH_3_ Losses

In this study, the results were divided into two topics for a better understanding of the treatments and for a fair comparison among the N-sources. The first topic includes the results of fertilizers applied in three split applications, and the second topic describes fertilizers applied in a single application. 

#### 2.2.1. Fertilizers Applied in Three Split Applications

The daily and accumulated losses of N-NH_3_ of the three fertilizer applications in each crop year were influenced (*p* ≤ 0.05) by the technologies for N-fertilizers. Except for urea+adhesive+CaCO_3_, all the technologies for N-fertilizers, ammonium nitrate, ammonium sulfate and stabilized fertilizers reduced N-NH_3_ losses compared to prilled urea. For the 2015/2016 crop season, the maximum losses or peaks of daily NH_3_ volatilization for prilled urea occurred 1.3 days after application (~5.8 kg N ha^−1^). For ammonium nitrate and ammonium sulfate, the maximum loss occurred 6.3 and 4.5 days after application, with values of 0.03 and 0.01 kg N ha^−1^, respectively. For fertilizers stabilized with Cu + B and NBPT, the maximum loss occurred at 4.9 and 2.6 days after application, with 1.0 and 2.5 kg N ha^−1^, respectively. Lastly, for urea dissolved in water and urea+adhesive+CaCO_3_, the maximum loss occurred at 1 and 1.15 days after application, with 1.5 and 1 kg N ha^−1^ (Table 1). In the 2015/2016 crop season, the mean accumulated losses in the first seven days were 9.2, 6.7, and 13% of the applied N for the first, second, and third split applications, respectively (Figure S1). In the 2016/2017 crop season, the maximum loss for prilled urea occurred two days after application, with a value of 7.4 kg N ha^−1^. For ammonium nitrate and sulfate, the maximum loss was at 13 and 5 days after application, with values similar to those of the first crop season (lower than 0.2 kg N ha^−1^). Urea + Cu + B and Urea + NBPT showed maximum losses at 3.7 and 4.3 days, with values of 4.0 and 3.2 kg N ha^−1^, respectively. Urea dissolved in water and urea + adhesive + CaCO_3_ had maximum losses at 1.5 and 1.9 days after N fertilization, with values of 1.6 and 10.3 kg N ha^−1^ (Table 2). In the 2016/2017 crop season, the mean general accumulated losses in the first seven days were 16, 8.5, and 11.7% for the first, second, and third split applications, respectively (Figure S3).

Regarding the accumulated N-NH_3_ losses in the 2015/2016 crop season, the mean value of losses was 10.6% of the applied N (average of the three split applications) (Table 3, Figure S5). For the treatments, the mean losses decreased as follows: Urea + adhesive + CaCO_3_ (25.5% of applied N) = prilled urea (23.2%) > urea + NBPT (13%) > urea + Cu + B (7.4%) > urea dissolved in water (4.5%) > ammonium sulfate (0.3%) = ammonium nitrate (0.2%). For the 2016/2017 crop season, the mean value was 13.7% (average of the three split applications). As for the treatments, the mean losses decreased in the following order: urea+adhesive+CaCO_3_ (30.3% of applied N) > prilled urea (24.2%) > urea + Cu + B (19.7%) > urea+NBPT (16%) > urea dissolved in water (4.5%) > ammonium sulfate (0.9%) = ammonium nitrate (0.8%) (Table 3, Figure S6). 

#### 2.2.2. Fertilizers Applied in a Single Application

In this section, the results of slow-release and controlled-release fertilizers and a blend will be presented. Here, fertilizers were applied in a single application, as they have the mechanism of gradual release of N to the soil. The results showed significant differences in N-NH_3_ losses by volatilization for both seasons. In the 2015/2016 crop season, the maximum loss occurred 35 days after the application for urea+elastic resin, with a mean value of 0.4 kg N ha^−1^. For the Blend N-fertilizer, at 24.7 days (0.19 kg N ha^−1^); urea-formaldehyde, at 7 days (0.08 kg N ha^−1^); urea+polyurethane, at 28.2 days (0.37 kg N ha^−1^). Regarding the 2016/2017 crop season, these N-sources behave similarly, with low values on the day of maximum loss. The urea+elastic resin treatment showed maximum loss at 40 days (~0.2 kg N ha^−1^); Blend N-fertilizer, at 9 days (~0.5 kg N ha^−1^); urea-formaldehyde, at 9 days (~0.04 kg N ha^−1^); and urea+polyurethane, at 31 days (0.22 kg N ha^−1^) (Table 4).

The losses accumulated by these sources in the 2015/2016 crop season were higher for the treatments: urea + polyurethane (6.4% of applied N) > urea + plastic resin (5.7%) = Blend N-fertilizer (4.6%) > urea-formaldehyde (0.6%) (Figure S2). In the 2016/2017 crop season, losses were higher for the Blend N-fertilizer (6.5% of applied N) = urea + plastic resin (5.9%) > urea + polyurethane (4%) > urea-formaldehyde (0.5%) (Table 5, Figure S4).

### 2.3. Summarizing Results of Ammonia Losses from N-Technologies 

Considering the way that the study was designed and conducted, it is not possible to compare the results of all technologies. However, a sequence of loss values presented by the sources can be established, considering the average of the two years of study. Thus, the decreasing order for the split treatments would be as follows: urea + adhesive + CaCO_3_ (27.9% of applied N = 84 kg N) > prilled urea (23.7% = 71 kg N) > urea + NBPT (14.5% = 43 kg N) = urea + Cu + B (13.5% = 40 kg N) > urea dissolved in water (4.2% = 12.6 kg N) > ammonium sulfate (0.6% = 1.8 kg N) = ammonium nitrate (0.5% = 1.5 kg of N). The decreasing order for the sources applied at a single time were: urea + elastic resin (5.8% = 17.4 kg N) = Blend N-fertilizer (5.5% = 16.6 kg N) = urea + polyurethane (5.2% = 15.6 kg N) > urea-formaldehyde (0.5% = 1.59 kg).

## 3. Discussion

In this study, the weather conditions greatly influenced N-NH_3_ losses by volatilization, particularly rainfall and temperature. In both coffee crop seasons, most N-NH_3_ losses occurred in the first seven days for the N-fertilizers applied in three split applications. The rainfall in these first days was essential for incorporating fertilizers into the soil and reducing N-NH_3_ emissions. Such a pattern was evidenced in both seasons.

In 2015/2016, the accumulated rainfall in the first seven days (19 mm) of the third split application was the lowest. Such low rainfall led to an increase of 40 and 90% in N-NH_3_ losses compared to the first and second split applications, respectively. The same pattern was not observed for the 2016/2017 season. However, an important issue must be considered: in the first and second N-fertilization, the mean NH_3_ losses in the first seven days were 16% and 8.5%, respectively. Such lower NH_3_ loss of 8.5% can be due to the absence of precipitation in the first two days of the first split application, which favored the permanence of the NH_4_^+^ from the N-fertilizers for a longer time on the soil surface. Regarding the pattern observed in the third split application, the losses were significant until the 13th day after fertilizer application, which is due to the lack of rainfall in the first days. These results show that rainfall before or after fertilization affects N losses by volatilization, particularly in the first seven days after the fertilizer application. Considering the initial seven days as the most critical phase to lose ammonia after applying prilled urea, the use of technologies associated with urea is critical to reducing N losses by volatilization [36,37]. In this context, NH_3_ losses depend on the volume and intensity of the rainfall [38].

This relationship between precipitation and N-fertilizer incorporation into the soil becomes even more complex in coffee plantations, as the architecture of the coffee plant restricts the direct incidence of rainfall, thus limiting the incorporation of the N-fertilizer applied in the canopy projection. Plant residues on the soil surface also function as a barrier to fertilizer incorporation (Figure S13). 

Regarding the efficiency of urea + NBPT in reducing N-NH_3_ losses by volatilization, it is possible that the NBPT inhibited the urease activity, which is responsible for urea hydrolysis [12,30]. The efficiency of the NBPT was evidenced by the delay of 1.3 days in daily ammonia volatilization peaks, the reduction in MDL by 63%, and the 38.8% reduction of the accumulated loss compared to prilled urea. Therefore, the NBPT effectively delayed the beginning of N-NH_3_ losses and reduced the accumulated losses compared to prilled urea. This delay possibly increased the chances of N-fertilizer incorporation by rainfall, which can reduce the losses of N by volatilization [39].

NBPT is currently the most used urease inhibitor worldwide [39]. A meta-analysis study reported that NBPT can reduce N-NH_3_ losses by 52% on average, compared to the ammonia losses of prilled urea [6]. Urease inhibitors are highly efficient in reducing N-NH_3_ losses by volatilization, but some aspects must be considered when NBPT is added to urea. These aspects include: its degradation under increased soil temperature [40], acidic soil pH [41], time and temperature of storage [12], and contact with phosphate fertilizers, which contain free acidity [42].

Urea + Cu + B reduced the N-NH_3_ losses by 68% and 18% compared to prilled urea in the 2015/2016 and 2016/2017 crop seasons. This efficiency in reducing losses is due to the potential for urease inhibition using Cu and B. The urease inhibition mechanism is due to the reaction of copper with the sulfhydryl groups of the urease enzyme, forming insoluble sulfites and inactivating the enzymatic action of urease [43,44]. Boric acid (H_3_BO_3_) can also inhibit urease activity but through a different inhibition mechanism. In this case, H_3_BO_3_ has a very similar structure to urea and functions as an analog substrate for ureases. Thus, it replaces almost perfectly the water molecules bound to Ni at the center of the reaction [45,46]. Urea treated with Cu and B is already commercialized in Brazil, and for this study, it was bought from the regional fertilizer market. Although Cu and B are potential urease inhibitors, the low concentrations found in some commercial products may not be enough to inhibit the enzyme [30]. Thus, proper concentrations must be evaluated in varying crops and cropping systems. Some issues related to the treatment process in the fertilizer industry still complicate the increase in the amounts of Cu and B added to urea, especially with the use of H_3_BO_3_, which has a low concentration of B (17%). 

Ammonia losses in the treatment urea + adhesive + CaCO_3_ were 18% higher than prilled urea in two coffee crop seasons. In this treatment, calcium carbonate was used as an alternative to elemental sulfur to create a physical barrier around the urea granule. In the present study, this technology was inefficient due to its limited effect as a physical barrier for the urea granule. CaCO_3_ increased the porosity and the contact with water enhanced its dissolution. This characteristic was evidenced when the day of the maximum NH_3_ loss was anticipated as well as the increase in the maximum NH_3_ daily loss in relation to the prilled urea. In addition, CaCO_3_ increases the alkalization that occurs around the urea granule hindering the pH buffering capacity of the region where the urea is hydrolyzed. Such a physical barrier with CaCO_3_ in urea increases the N-NH_3_ losses. Furthermore, this concept was also verified by the NH_3_ accumulated losses from the da urea + Adhesive + CaCO_3_, which was higher than the prilled urea. Thus, we concluded that the alkaline (CaCO_3_) coating urea was inefficient to reduce the ammonia losses by volatilization.

There are two N-fertilizers widely used in Brazilian coffee plantations, namely ammonium nitrate and ammonium sulfate. In this study, the reduction of N-NH_3_ losses for these two sources was higher than 97% in both crop seasons. The irrelevant NH_3_ losses from these N-sources are related to their acidic-to-neutral reaction in soil, mainly at pH < 7 [47]. Another positive aspect is that these fertilizers do not depend on weather conditions at the moment of their application. Thus, both ammonium nitrate and sulfate can be smart options for N-fertilization in coffee crop systems.

Altogether, the application of urea dissolved in water by drench draws attention to the technologies used to mitigate prilled urea losses by volatilization. In the present scientific study, this treatment showed good efficiency in reducing N-NH_3_ losses by volatilization. The days of maximum loss occur very similarly to prilled urea applied on the soil surface. However, the losses in these days of maximum loss are, on average, 3.8 and 4.5 times lower than prilled urea for the 2015/2016 and 2016/2017 crop seasons, respectively. Moreover, the accumulated losses of urea dissolved in water were five and six times lower than those observed for prilled urea in the 2015/2016 and 2016/2017 seasons, respectively. Such reduced losses are due to the dissolution of urea in water, which percolates to subsurface layers in the soil carrying the urea molecules, thus reducing N-NH_3_ losses by volatilization. For this treatment, no additive was added to the conventional urea. However, urease or nitrification inhibitors can also be added to the urea solution [48], thus improving urea use efficiency, that is, the technologies available in the market can be associated with strategies that can further increase the N use efficiency. 

Considering the management of coffee plantations in Brazil, the application of urea solution can be performed along with systemic insecticides. Such products are applied directly in the ground, under the projection of the coffee tree canopy. However, it is important to evaluate the compatibility between the products to be applied, as well as the spray volume used and its relationship with the urea concentration in the solution, related to the solubility product constant [16]. Another strategy would be to add micronutrients to the urea solution in order to standardize the distribution. In addition, increasing the concentration of B and Cu in the urea solution could inhibit urease activity and help mitigate ammonia losses. 

In this study, it was not possible to compare the conventional, stabilized, slow-, and controlled-release fertilizers. However, the latter treatments showed interesting patterns when applied to coffee cropping systems. The basis of slow-release, controlled-release, and Blend N-fertilizers is urea, but the associated technologies lead to contrasting responses in N-NH_3_ losses. The average accumulated loss by those sources is lower than 6% of the applied N when averaging the two crop seasons. 

This pattern observed for controlled-release fertilizers (urea + elastic resin, urea + polyurethane) is explained by the way N is released into the soil. The release of N in controlled-release fertilizers occurs by the diffusion of urea from inside the granule through the coating into the soil solution. This process starts with increasing steam pressure and water intake into the granule. Then, osmotic pressure inside the capsule increases and creates a diffusion gradient from the fertilizer to the soil solution [49]. The gradual release reduces the excess of N-mineral available in the soil solution, which is susceptible to volatilization, denitrification, and leaching. Controlled-release urea improves the synchronism between the N release from fertilizer granules and its absorption by the plants, thus reducing N losses and improving nitrogen use efficiency in coffee crop environments [2,50]. 

The chemical reactions in the Urea-Formaldehyde production process reduce the nitrogen solubility in water compared to conventional sources of N, owing to the formation of long and short polymerization chains. This reduced solubility has varying effects on the rates of N release over time. The methylene urea chains formed in the Urea-Formaldehyde production depend on the activity of microorganisms in a process similar to N mineralization in the soil. Such a process prevents all the N from being readily released into the soil and is thus subject to the transformations needed to produce NH_3_ [51,52,53,54,55]. From an agronomic perspective, the lower N-NH_3_ losses are due to the reduction of excessive mineral N in the soil solution, which is susceptible to N-losses. The same pattern was also observed in the controlled-release urea. In the present study, the release time of controlled-release or slow-release fertilizers such as urea-formaldehyde was not verified. However, these enhanced efficiency fertilizers should be further investigated regarding their potential for proper N supply for coffee crop systems.

The Blend N-fertilizer, a blend of urea stabilized with NBPT and urea coated with elemental sulfur and polymer, was also efficient in reducing N losses, which did not exceed 7% in both coffee crop seasons. In this blend, part of the urea is in the soluble form and is protected by the NBPT as a urease inhibitor. The Blend N-fertilizer improves the N provision to the coffee plants over time as it combines the fast release of the soluble urea mixed with NBPT and the controlled-release urea to provide nitrogen for a longer time. The N-NH_3_ losses were similar to the 100% coated treatments compared to blend N-fertilizers, thus demonstrating the efficiency of this technology to supply N to the coffee plant.

### Highlights of Economic View of N-Fertilizers Technologies 

From an economic perspective, here we present a short overview related to the prices of N-fertilizer technologies assessed in this scientific paper. Prilled urea has the lowest cost in the market, considering its increased N concentration and disregarding the high N-NH_3_ losses. In sequence, are fertilizers stabilized with NBPT, Cu, and B, which have similar market values, followed by urea + adhesive + CaCO_3_. The prices of Blend-N-fertilizer reduce as the proportion of the stabilized and conventional ureas increase in the physical mixture and their prices are higher than conventional and stabilized N-fertilizers. In addition, Blend-N-fertilizers have a lower cost compared to 100% of controlled-release urea. 

In this context, urea + polyurethane and urea + plastic resin, which are controlled-release or added-value fertilizers, have similar market prices. In addition, the prices of controlled-release fertilizers may vary according to the material used in the coating and the thickness of the coating. Finally, urea-formaldehyde as well as controlled-release fertilizers require investments in industrial processing such as infrastructure with specific conditions to produce these added-value N-fertilizers. In some situations, in Brazil, urea-formaldehyde has been used as a blended form with conventional urea and/or ammonium sulfate reducing its price compared to pure urea-formaldehyde. In general, pure urea-formaldehyde has similar prices to pure controlled-release fertilizers.

From an agronomic/economic point of view, the decision on which N sources would be interesting for application in coffee plantations must consider the costs of the fertilizer application. Fertilization with conventional and stabilized fertilizers must be split into three or more applications. Slow- and controlled-release fertilizers can be applied in a single operation reducing the costs (labor, fuels, machine maintenance, and depreciation), time of mechanized operation on the farm, and soil compaction due to the reduction of N splits compared to conventional and stabilized fertilizers. On the other hand, the Blend N-fertilizer technology is more expensive than conventional and stabilized fertilizers, but almost always has a lower value compared to slow- and controlled-release fertilizers. Besides, Blend N-fertilizers provide better synchronism between the N release and its absorption by the plants. 

## 4. Materials and Methods

### 4.1. Characterization of the Experimental Area

**Figure 1:**
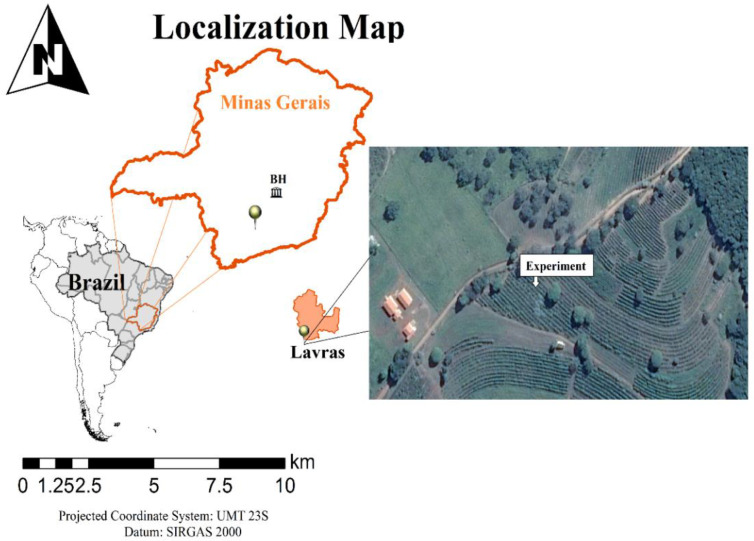
Location map of the experimental areas in Lavras, Minas Gerais, Brazil.

The experiment was conducted in coffee plantations under field conditions for two crop seasons, 2015/2016 and 2016/2017, in Lavras, Minas Gerais (MG), Brazil (Figure 1). Lavras (910 m a.s.l., 21°14′06″ S 45°00′00″ W) is located in a traditional region of coffee production in Brazil, within the Campos das Vertentes geographical indication. According to Köppen’s classification, the climate is Cwa, mesothermal with mild summers and dry winters. The mean annual precipitation is approximately 1472 mm, the mean annual temperature is 19.4 °C [56].

The coffee plantation in the production phase was planted with the “Catuaí Vermelho” cultivar, line 144. At the beginning of the experiment, the plantation was six years old. The spacing used in the planting was 3.7 m between rows and 0.7 m between plants, totaling 3861 plants ha^−1^.

The soil was classified as “Latossolo Vermelho Amarelo Distroférrico (LVdf)” according to the Brazilian System of Soil Classification [57], or Haplustox [58]. Before installing the experiment, soil samples were collected at the 0–0.2 m depth for soil texture [59] and soil chemical analyses. (Table 6) lists the result of the soil chemical analysis and texture.

### 4.2. Experimental Design

In this study, different sources of N-fertilizers were used, which were applied in a single application or split into three applications. Thus, two different group experiments were carried out in the same area, but the management practices (other than fertilization) remained similar. The experiments were designed as follows: Group 1) seven treatments, consisting of conventional and stabilized fertilizers and urea dissolved in water (management strategy) the experimental design in the field was randomized blocks with three repetitions, totaling 21 plots; and Group 2) four treatments, consisting of slow-, controlled-, and blend fertilizers, the experimental design in the field was randomized blocks with three repetitions, totaling 12 plots. For conventional and stabilized fertilizers, a dose of 300 kg ha^−1^ was divided into three applications. For the other treatments (Group 2) a dose of 300 kg ha^−1^ was applied in a single application. The treatments will be described in detail in the next topic. Each experimental unit consisted of 14 coffee plants. The ten central plants comprised a useful area for data collection.

### 4.3. Characterization of the Fertilizers

The fertilizers used in this study were chosen based on technologies used in Brazilian coffee production systems. They were divided into four groups and characterized according to the type of technology used. We photographed all fertilizers with a Canon camera, SL3 DSLR model, and an Olympus microscope, SZ60 Japan model. Fertilizers classified as controlled-release were characterized by scanning electron microscopy (SEM) and energy dispersion X-ray spectroscopy (EDS). 

The first group included the conventional fertilizers: (1) prilled urea (45% N), (2) Ammonium nitrate (31% N), and (3) Ammonium sulfate (21% N and 24% S-SO_4_^2-^). Another treatment containing prilled urea (45% N) diluted in water at a concentration of 50 g L^−1^ was also added, aiming to reduce N-NH_3_ losses and to completely dissolve urea: (4) urea dissolved in water. 

Another group amongst the technologies used in this study was the stabilized fertilizers, which have additives that can inhibit or delay some process of N transformation in the soil: (5) urea treated with Cu and B (44% N; 0.4% B as boric acid and 0.15% Cu as copper sulfate) and (6) urea treated with NBPT (45% N). This group of fertilizers consists of urease inhibitors (NBPT, NPPT, Cu, and B). The functioning of Cu and B as urease inhibitors depends on the concentrations added to the fertilizer. Besides being micronutrients, Cu and B have competitive and non-competitive urease inhibition capacities, respectively [60]. 

The group of controlled-release fertilizers was also included in this study: (7) urea coated with elastic resin (44% N and 43.8 µm of average coating thickness), (Figure S8) (8) urea coated with polyurethane (40% N and 56.4 µm of average coating thickness), (Figure S9). 

The group of chemically modified or slow-release fertilizers was represented in this study by (9) urea-formaldehyde (26% N). This product results from the reaction of formaldehyde molecules (H_2_CO) with urea (NH_2_)_2_CO under controlled temperature and pressure. This reaction forms chains of C and N with different sizes and degrees of polymerization. 

A treatment for physical protection of the urea granules was included in this study: (10) urea + adhesive + CaCO_3_. This treatment included a compound that agglutinates calcium carbonate (CaCO_3_), creating a physical barrier of adhesive and CaCO_3_. This barrier temporarily prevents contact between the soluble conventional urea and soil moisture. 

Lastly, a fertilizer based on the physical mixture of technologies (blend) was added to the present study, constituting the treatment called: (11) Blend N-Fertilizer (39% N, 9% S^0^) (Figure S7). In this case, the release of N to the system occurs in different stages, as this blend is a mixture of conventional urea with a controlled release fertilizer (urea coated with elemental sulfur (S^0^) + polymer), measuring 67.5 µm of coating thickness and a stabilized fertilizer (urea treated with NBPT, most of the times). Therefore, the blend aims at the synchronization of the release of N by the fertilizer and its absorption by the plant, which reduces N excess in the system and N-NH_3_ losses by volatilization.

For the other treatments, the granulometry of the fertilizers varied from 1 to 4 mm, as officially specified by the Brazilian legislation for granulated fertilizers. Further physical characteristics of the treatments can be found in figures (Figures S7–S12). 

The treatments used in this study were applied at the 300 kg N ha^−1^ dose per year. The application of the treatments prilled urea, ammonium nitrate, ammonium sulfate, stabilized (urea + NBPT and urea + Cu + B) and urea + adhesive + CaCO_3_ were split into three doses of 100 kg N ha^−1^ into the two crop seasons of the experiment. Urea dissolved in water was applied via drench at a dose of 1.6 L m^−1^, totaling 16 L per plot, following the same criteria described for the split application. The slow-release, controlled-release, and blend fertilizers were applied at a single dose of 300 kg N ha^−1^ per year. All fertilizers were applied as topdressing, superficially, and under the canopy projection of the coffee plants. The applications for the 2015/2016 season were conducted on 6 November 2015, 11 January, and 10 March 2016. The 2016/2017 season received the applications following the same interval. The treatments received the slow-release, controlled-release, and blend-N fertilizers on the same day as the first application of the conventional and stabilized fertilizers.

### 4.4. Complementary Management of Soil Fertility

Liming was performed 60 days before applying the N fertilizers in each treatment plot, aiming to increase soil base saturation to 60%. The dose of 2 t ha^−1^ of agricultural lime (PRNT 100%) was used in both crop seasons. Maintenance fertilization was performed with potassium chloride (KCl—60% K_2_O) and simple superphosphate (SFS—20% P_2_O_5_) fertilizers, applied at doses of 300 kg K_2_O ha^−1^ per year and 100 kg P_2_O_5_ ha^−1^ per year, respectively, under the canopy projection of the coffee plants [61].

The micronutrients were applied via leaf fertilization along with phytosanitary control. These procedures were performed both during the formation period of the coffee plantation and over the experimental period. A total of 5 kg ha^−1^ of a commercial product containing the following nutrients were applied: 6.0% zinc (ZnSO_4_), 3.0% boron (H_3_BO_4_), 2.0% manganese (MnSO_4_), 10.0% copper (Cu (OH)_2_), 10.0% sulfur, 1.0% magnesium (MgSO_4_) and 10.0% K_2_O (KCl). The spray volume applied was 300 L ha^−1^, totaling three applications per year in 45-day intervals between November and February each year.

### 4.5. Quantification of N-NH_3_ Losses

**Figure 2:**
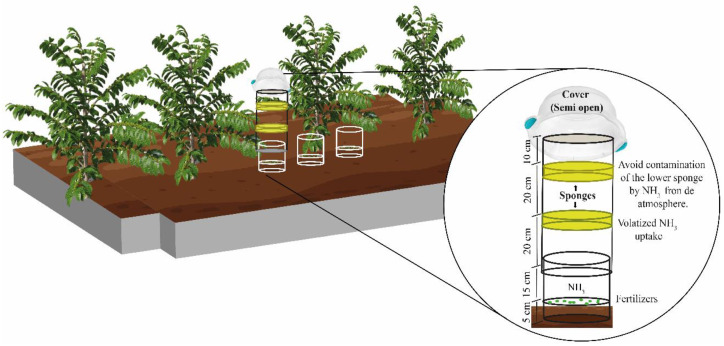
Illustration of the collectors used in the quantification of ammonia losses.

The losses of N-NH_3_ owing to the application of N fertilizers were quantified using the semi-open collector adapted by Lara Cabezas [62]. In the first year, three PVC bases (0.2 m height and 0.2 m diameter) were installed 30 days before the application of the fertilizers in each experimental plot, under the canopy projection of the coffee plants, and at a depth of 0.05 m into the soil (Figure 2). These PVC chambers were kept in the field during the two years of the experiment. 

Collection chambers were made in PVC with a diameter similar to the bases. The chambers had lids that prevented water to enter, but allowed air circulation. They had 0.5 m height and specifications as described in (Figure 2). The amount of fertilizer corresponding to the dose applied per hectare was added within each base (0.2 linear meters). To calculate the dose of N, we considered the useful distance in linear meters of one hectare and the 3.7 m spacing between lines, totaling 2702.7 m. The dose of N was corrected for the equivalent base diameter (0.20 m). For the N-sources whose fertilization was split into three applications, 7.4 g of N was added to each base on the same day that the fertilization of the plots was performed. As for the treatments that received one single fertilizer application, 22.20 g of N was added to each base. The collection chamber was added to one of the bases, in all plots, immediately after applying fertilizers on the bases.

Two laminated foam discs with 0.02 g cm^−3^ density, 0.2 m thickness, and the same diameter as the PVC tubes were placed inside each semi-open collector. The foam discs were soaked with 80 mL of phosphoric acid (H_3_PO_4_; 60 mL L^−1^) solution and glycerin (50 mL L^−1^). The lower disc was placed inside the chamber at a height of 0.35 m from the soil, and the upper disc at 0.2 m from the lower one (Figure 2). The lower foam disk aimed to capture the ammonia released by the treatments, as the upper disk aimed to avoid contamination of the lower disk by N-NH_3_ released from the rest of the fertilized line.

Foam disks were collected on the 1st, 2nd, 3rd, 4th, 5th, 7th, 9th, 12th, 15th, 19th, 24th, and 31st days after the application of fertilizers in the 2015/2016 crop season for conventional and stabilized fertilizers (Group 1). In 2016/2017, the collections were performed on the 1st, 2nd, 3rd, 4th, 5th, 7th, 9th, 12th, 14th, 17th, and 20th days, and until the 34th day after applying the treatments. The collections in the treatments with slow- and controlled-release fertilizers (Group 2) were performed on the same day as the conventional and stabilized fertilizers. However, they were extended until the 208th day of the first crop season and until the 235th of the second crop season.

After each sponge change, the chamber was rotated from one base to another to consider the influence of the spatial variability of ammonia emission. This rotation allows a greater influence on climatic variations, such as temperature and precipitation.

The solution in the sponges collected from the field was extracted by filtration in a Büchner funnel connected to a vacuum pump. The extraction was performed after ten sequential washes with 40 mL of deionized water each. The extracts were stored in a cold chamber for a maximum period of 5 days, and after that, they were analyzed. From the extract, 20 mL aliquots were taken to determine the N content by distillation by the Kjeldahl method [63]. The N content in the sample was calculated according to equation 1, adapted from Nogueira and Souza [64]: TN = [(Va − Vb) × F × 0.1 × 0.014 × 100]/P1, in which, TN: Total N concentration in the sample (%), Va: Volume of hydrochloric acid solution spent on the sample titration (mL), Vb: Volume of hydrochloric acid solution spent on the blank titration (mL), F: Correction factor for 0.01 M hydrochloric acid, P1: Sample mass (g).

The values obtained in the N content calculations referred to the area occupied by the base of the chambers installed in the field. These values were then extrapolated to the percentage of loss of N-NH_3_ per hectare. The accumulated losses in the assessment were calculated by adding the losses from the 1st to the 2nd day, then adding this value to the losses of the 3rd day, and so on until the last day of collection.

### 4.6. Statistical Analysis

The treatments were submitted to a non-linear regression analysis using a logistic model (equation 2) to evaluate the ammonia loss by volatilization: Yi = [α/1 + e^k^ (b − daai)] + Ei, in which, Yi is the i-th observation of the accumulated loss of N-NH_3_ in %, being i = 1, 2, …, n; daai is the i-th day after the application of the treatment; α is the asymptotic value that can be interpreted as the estimation of the maximum accumulated loss of N-NH_3_; *b* is the abscissa of the inflection point and indicates the day of the maximum loss by volatilization; k is the value that represents the precocity index, and the higher its value, the lower the time needed to reach the maximum loss by volatilization (*α*); Ei is the error associated to the i-th observation, which is assumed to be independent and equally distributed according to a zero average standard and constant variance, E ~ N (0, I σ2).

This model is already used to estimate plant growth but has recently been applied to estimate the N-NH_3_ accumulated loss [6,65,66].

To estimate the maximum daily loss (day when the highest N-NH_3_ loss occurred), that is, to determine the inflection point of the curve, the following equation was used: MDL = k × (α/4), in which, k is a relative index used to obtain to a maximum daily loss of ammonia (MDL), and α is the asymptotic value that can be interpreted as the maximum amount of accumulated N-NH_3_ loss. The “nlme” package was used in the modeling of the N-NH_3_ losses data, using the R 3.3.1 software [67].

Normality and homoscedasticity of the data were verified by the Shapiro-Wilk and Bartlett tests, respectively. Then, an analysis of variance was performed to test the influence of the N sources on the N-NH_3_ losses by volatilization. The significance of the differences was evaluated at *p* < 0.05. After validating the statistical model, the mean values were grouped by the Scott-Knott algorithm using the R 3.3.1 software [67].

## 5. Conclusions

Nitrogen fertilizers such as conventional urea can be used to improve nutrient use efficiency in coffee production environments by using technologies such as urease inhibitors and polymer coatings. Altogether, conventional urea had ammonia losses equal to 24% of N applied to promote lower N-use efficiency during two coffee seasons. Calcium carbonate as a physical coating around the urea granules performed poorly compared to all the other N-fertilizer technologies with ammonia volatilization losses 18% greater than conventional urea. Urea dissolved in water is an interesting N-fertilization management strategy for coffee farmers as the ammonia losses were only 4.2% of the applied N. Urea stabilized with N-(n-butyl) thiophosphoric triamide (NBPT) is a useful industrial innovative technology to mitigate ammonia losses because urease inhibitor as additive reduces ammonia losses by 39%. Slow- and controlled-release urea and Blend N-fertilizer are interesting added-value N-fertilizers to improve coffee crop nutrition over time because they can be applied in a single mechanized operation with ammonia losses lower than 7% of the applied N. Conventional N-fertilizers such as ammonium nitrate and ammonium sulfate showed negligible ammonia losses demonstrating its potential as interesting choices in comparison with conventional urea to mitigate ammonia emissions. Also, they can be applied regardless of soil humidity and climate conditions. In summary, in this scientific paper, we presented some highlights of cutting-edge technologies as a plan for the efficient use of N-fertilizers in coffee crop production environments. However, our research group is engaged in similar studies in the coffee crop, where not only aspects related to ammonia loss are being evaluated, but also emission of the other GHG, soil enzyme activity, and aspects related to plant nutrition, thus allowing better understanding of the N cycle for the coffee plant.

## Figures and Tables

**Table 1:** Regression parameters adjusted for the accumulated and maximum daily losses of N-NH_3_ from conventional and stabilized N fertilizers in the 2015/2016 crop season.

Treatment	Split Fertilization	Parameters				MDL (kg)
		α	b	k	R^2^	
		Maximum NH_3_ Loss	Day of the Maximum Loss			
Prilled urea	1	23.65	1.31	1.45	0.97	8.573
	2	13.97	0.77	1.24	0.99	4.331
	3	30.78	1.70	0.59	0.99	4.540
Ammonium nitrate	1	0.26	6.70	0.27	0.94	0.018
	2	0.21	4.89	0.64	0.95	0.034
	3	0.27	7.21	0.40	0.94	0.027
Ammonium sulfate	1	0.57	0.12	0.22	0.97	0.031
	2	0.06	6.79	0.17	0.95	0.003
	3	0.31	6.54	0.08	0.98	0.006
Urea + NBPT	1	9.97	2.72	0.40	0.98	0.997
	2	6.33	2.20	1.52	0.98	2.405
	3	22.32	2.78	0.73	0.98	4.073
Urea dissolved in water	1	7.19	0.89	1.80	0.96	3.236
	2	2.45	−7.19	0.23	0.94	0.141
	3	3.80	0.70	1.32	0.97	1.254
Urea + adhesive + CaCO_3_	1	22.67	1.23	2.49	0.96	14.112
	2	21.89	1.21	2.35	0.99	12.860
	3	30.41	1.05	0.56	0.98	4.257
Urea + Cu + B	1	3.46	5.32	0.40	0.98	0.346
	2	2.04	1.29	2.22	0.98	1.132
	3	16.34	7.98	0.39	0.99	1.593

α: Asymptotic value (percentage of estimated maximum accumulated loss); b: Day when the maximum ammonia loss occurs; k: relative index; MDL (maximum daily loss of ammonia) and NBPT: N-(n butyl) thiophosphoric triamide.

**Table 2:** Regression parameters adjusted for the accumulated and maximum daily losses of N-NH_3_ from conventional and stabilized N fertilizers in the 2016/2017 crop season.

Treatment	Split Fertilization	Parameters				MDL
		α	b	k	R^2^	
		Maximum NH_3_ Loss	Day of the Maximum Loss			(kg)
Prilled urea	1	32.03	1.72	1.32	0.99	10.570
	2	16.76	2.40	0.85	0.96	3.562
	3	22.28	1.97	1.46	0.98	8.132
Ammonium nitrate	1	0.48	9.68	0.22	0.94	0.026
	2	0.51	24.80	0.07	0.81	0.009
	3	1.72	4.90	0.43	0.93	0.185
Ammonium sulfate	1	0.05	2.32	1.10	0.91	0.014
	2	0.36	4.21	0.52	0.91	0.047
	3	1.65	8.37	0.33	0.97	0.136
Urea + NBPT	1	18.67	3.01	1.06	0.99	4.948
	2	10.41	5.25	0.79	0.97	2.056
	3	17.32	4.73	0.59	0.98	2.555
Urea dissolved in water	1	5.66	1.69	0.90	0.99	1.274
	2	4.77	1.45	2.74	0.94	3.267
	3	1.33	1.43	1.24	0.93	0.412
Urea + adhesive + CaCO_3_	1	36.22	1.70	1.81	0.99	16.390
	2	18.56	1.78	1.16	0.93	5.382
	3	34.12	2.10	1.09	0.98	9.298
Urea + Cu + B	1	20.27	3.54	1.31	0.98	6.638
	2	15.29	2.84	0.73	0.95	2.790
	3	21.58	4.79	0.54	0.98	2.913

α: Asymptotic value (percentage of estimated maximum accumulated loss); b: Day when the maximum ammonia loss occurs; k: relative index; MDL (maximum daily loss of ammonia) and NBPT: N-(n butyl) thiophosphoric triamide.

**Table 3:** Mean accumulated losses of ammonia (% of applied N), for conventional and stabilized N fertilizers, in three fertilizations in the coffee plantation, during the 2015/2016 and 2016/2017 crop seasons.

Treatment	Ammonia Loss (%)								Mean ** (%)	PCRDU ** (%)
	Season 2015/2016				Season 2016/2017					
	1st	2nd	3rd	Mean	1st	2nd	3rd	Mean		
Prilled urea	24.2a	14.0b	31.6a	23.2a	32.3a	17.3a	23.1b	24.2b	23.7b	-
Urea dissolved in water	7.3b	2.4c	4.0d	4.5d	5.6c	5.1b	1.5c	4.0e	4.2d	82.3
Ammonium sulfate	0.6c	0.1c	0.3d	0.3e	0.6c	0.4b	1.9c	0.9e	0.6e	97.5
Ammonium nitrate	0.3c	0.2c	0.3d	0.2e	0.5c	0.3b	1.8c	0.8e	0.5e	97.9
Urea + Cu + B	3.5c	2.0c	16.7c	7.4c	20.7b	15.8a	22.8b	19.7c	13.5c	43
Urea + adhesive + CaCO_3_	23.7a	22.0a	31.0a	25.5a	36.7a	19.3a	35.1a	30.3a	27.9a	−17.7 ***
Urea + NBPT	9.9b	6.4c	22.7b	13.0b	18.8b	11.3a	18.0b	16.0d	14.5c	38.8
Mean	9.9	6.7	15.2	10.6	16.4	9.9	14.9	13.7	12.1	56.9
Coefficient of Variation	18	17.9	16.2	11.7	15.4	27.9	11.2	18.2	8.5	-

NBPT: N-(n butyl) thiophosphoric triamide. Note: In each crop season, 300 kg N ha^−1^ per year were split into three equal applications for conventional and stabilized N fertilizers, totaling 600 kg N ha^−1^ for both crop seasons. Means followed by the same lowercase letter in the column do not differ by the Scott-Knott test (*p* ≤ 0.05). Mean of the six fertilization sources performed between November and February during both seasons ** (PCRDU) Percentage change decrease compared to Prilled Urea. *** Negative value indicates increased volatilization compared to prilled urea.

**Table 4:** Regression parameters adjusted for the accumulated and maximum daily losses of N-NH_3_ from slow-release and controlled-release N fertilizers in the 2015/2016 and 2016/2017 crop seasons.

Treatment	Crop Season	Parameters				MDL
		α	b	k	R^2^	
		Maximum NH_3_ Loss	Day of the Maximum Loss			(kg)
Urea + elastic resin	2015/2016	5.67	35.94	0.10	0.99	0.425
Blend N-fertilizer		4.29	24.71	0.06	0.94	0.193
Urea-formaldehyde		0.53	6.99	0.20	0.82	0.080
Urea + polyurethane		6.20	28.25	0.08	0.98	0.372
Urea + elastic resin	2016/2017	5.75	40.56	0.05	0.99	0.216
Blend N-fertilizer		6.07	9.23	0.12	0.95	0.546
Urea-formaldehyde		0.42	8.88	0.14	0.90	0.044
Urea + polyurethane		3.80	31.10	0.08	0.99	0.228

α: Asymptotic value (percentage of estimated maximum accumulated loss); b: Day when the maximum ammonia loss occurs; k: relative index; MDL (maximum daily loss of ammonia) and NBPT: N-(n butyl) thiophosphoric triamide.

**Table 5:** Mean accumulated losses of ammonia (% of applied N), for slow-release and controlled-release N fertilizers, in one single application in the coffee plantation, during the 2015/2016 and 2016/2017 crop seasons.

Treatment	Crop Season						Mean of Two Crop Seasons **
	Ammonia Loss (%)						
	2015/2016			2016/2017			
	1st	2nd	3rd	1st	2nd	3rd	**(%)**
Blend N-fertilizer	4.59a			6.46a			5.53a
Urea + elastic resin	5.71a			5.91a			5.81a
Urea-formaldehyde	0.58b			0.48c			0.53b
Urea + polyurethane	6.40a			4.02b			5.21a
Mean	4.32			4.22			4.22
Coefficient of Variation	0.43			0.47			0.47
Precipitation (mm)	694 *			455 *			574 ***

Note: In each crop season, 300 kg N ha^−1^ per year were applied into one single application for slow and controlled-release N fertilizers, totaling 600 kg N ha^−1^ for both crop seasons. Means followed by the same lowercase letter in the column do not differ by the Scott-Knott test (*p* ≤ 0.05). * Sum of precipitation during the evaluation periods, which were 208 and 235 days in the 2015/2016 and 2016/2017 crop seasons, respectively. ** Mean of the six fertilizations performed between November and February of each crop season/year. *** Mean of precipitation during the evaluation periods, which were 208 and 235 days in the 2015/2016 and 2016/2017 crop seasons.

**Table 6:** Chemical characterization and soil texture of the experimental area at the 0–20 cm depth, before the application of the treatments.

pH	K	P	Cu	B	Ca^2+^	Mg^2+^	Al^3+^	CEC	OM	BS	Sand	Silt	Clay
	mg dm^−3^				cmol_c_ dm^−3^				%				
4.6	92	16	1.5	0.3	1.7	0.4	0.7	11.4	2.4	30	18	24	58

pH in water (1:2.5); P, K, and Cu extracted by Mehlich-1; B extracted by hot water; Ca^2+^, Mg^2+^, and Al^3+^ extracted by 1 M KCl; CEC = Cation Exchange Capacity at pH 7.0; OM = soil organic matter; BS = base saturation; Sand, silt, and clay = particle-size fractions.

## Data Availability

Not applicable.

## Supplementary Materials

The following supporting information can be downloaded at: https://www.mdpi.com/article/10.3390/plants11233323/s1, Figure S1: Daily N-NH_3_ losses by volatilization in the first, second, and third split application (a, b, and c) of conventional and stabilized N fertilizers. Rainfall, average temperature, and relative air humidity after splitting the N fertilization in the 2015/2016 crop season (d); Figure S2: Daily (a) and accumulated (b) N-NH_3_ losses by volatilization in slow- and controlled-release N fertilizers. Rainfall, average temperature, and relative air humidity after splitting the N fertilization in the 2015/2016 crop season (c); Figure S3: Daily N-NH_3_ losses by volatilization in the first, second, and third split application (a, b, and c) of conventional and stabilized N fertilizers. Rainfall, average temperature, and relative air humidity after splitting the N fertilization in the 2016/2017 crop season (d); Figure S4: Daily (a) and accumulated (b) N-NH_3_ losses by volatilization in slow- and controlled-release N fertilizers. Rainfall, average temperature, and relative air humidity after splitting the N fertilization in the 2016/2017 crop season (c); Figure S5: Accumulated N-NH_3_ losses by volatilization in the first, second, and third split application (a, b, and c) of conventional and stabilized N fertilizers in the 2015/2016 crop season; Figure S6: Accumulated N-NH_3_ losses by volatilization in the first, second, and third split application (a, b, and c) of conventional and stabilized N fertilizers in the 2016/2017 crop season; Figure S7: Thickness of surface coatings by MEV images (a) and elemental composition of surface coatings (b) of the controlled release source: urea coated with elemental sulfur (S^0^) + polymer (Blend N—fertilizer); Figure S8: Thickness of surface coatings by MEV images (a) and elemental composition of surface coatings (b) of the controlled release source: urea coated with plastic resin; Figure S9: Thickness of surface coatings by MEV images (a) and elemental composition of surface coatings (b) of the controlled release source: urea coated with polyurethane; Figure S10: Images of the conventional N fertilizers: urea (a), ammonium nitrate (b), and ammonium sulfate (c); Figure S11: Images of the stabilized N fertilizers: urea treated with NBPT (a) and urea treated with Cu and B; Figure S12: Images of the fertilizers: Slow-release: Urea-formaldehyde (a) and Physical barrier: Urea + adhesive + CaCO_3_ (b); Figure S13: Dry leaves in the canopy projection of the coffee plant hindering fertilizer incorporation.
