# Supplementary material for: Technologies for Fertilizers and Management Strategies of N-Fertilization in Coffee Cropping Systems to Reduce Ammonia Losses by Volatilization
Source: Plants (Basel). 2022 Dec 1;11(23):3323. doi: 10.3390/plants11233323 (PMC9741429; doi:10.3390/plants11233323)
Supplement: Supplementary file 1 [file plants-11-03323-s001.zip › plants-1964999-supplementary.pdf]

Technologies for fertilizers and management strategies of N-fertilization in coffee cropping systems to reduce ammonia losses by volatilization

Supplementary Material

Figure S1. Daily N-NH_3 losses by volatilization in the first, second, and third split application (a, b, and c) of conventional and stabilized N fertilizers. Rainfall, average temperature, and relative air humidity after splitting the N fertilization in the 2015/2016 crop season (d).

Figure S2. Daily (a) and accumulated (b) N-NH_3 losses by volatilization in slow- and controlled-release N fertilizers. Rainfall, average temperature, and relative air humidity after splitting the N fertilization in the 2015/2016 crop season (c).

Figure S3. Daily N-NH_3 losses by volatilization in the first, second, and third split application (a, b, and c) of conventional and stabilized N fertilizers. Rainfall, average temperature, and relative air humidity after splitting the N fertilization in the 2016/2017 crop season (d).

Figure S4. Daily (a) and accumulated (b) N-NH_3 losses by volatilization in slow- and controlled-release N fertilizers. Rainfall, average temperature, and relative air humidity after splitting the N fertilization in the 2016/2017 crop season (c).

Figure S5. Accumulated N-NH_3 losses by volatilization in the first, second, and third split application (a, b, and c) of conventional and stabilized N fertilizers in the 2015/2016 crop season.

Figure S6. Accumulated N-NH_3 losses by volatilization in the first, second, and third split application (a, b, and c) of conventional and stabilized N fertilizers in the 2016/2017 crop season.

Figure S7. Thickness of surface coatings by MEV images (a) and elemental composition of surface coatings (b) of the controlled release source: urea coated with elemental sulfur (S^0) + polymer (Blend N – fertilizer).

Figure S8. Thickness of surface coatings by MEV images (a) and elemental composition of surface coatings (b) of the controlled release source: urea coated with plastic resin.

Figure S9. Thickness of surface coatings by MEV images (a) and elemental composition of surface coatings (b) of the controlled release source: urea coated with polyurethane.

Figure S10. Images of the conventional N fertilizers: urea (a), ammonium nitrate (b), and ammonium sulfate (c).

Figure S11. Images of the stabilized N fertilizers: urea treated with NBPT (a) and urea treated with Cu and B.

Figure S12. Images of the fertilizers: Slow-release: Urea-formaldehyde (a) and Physical barrier: Urea + adhesive + $CaCO_3$ (b).

Figure S13. Dry leaves in the canopy projection of the coffee plant hindering fertilizer incorporation.

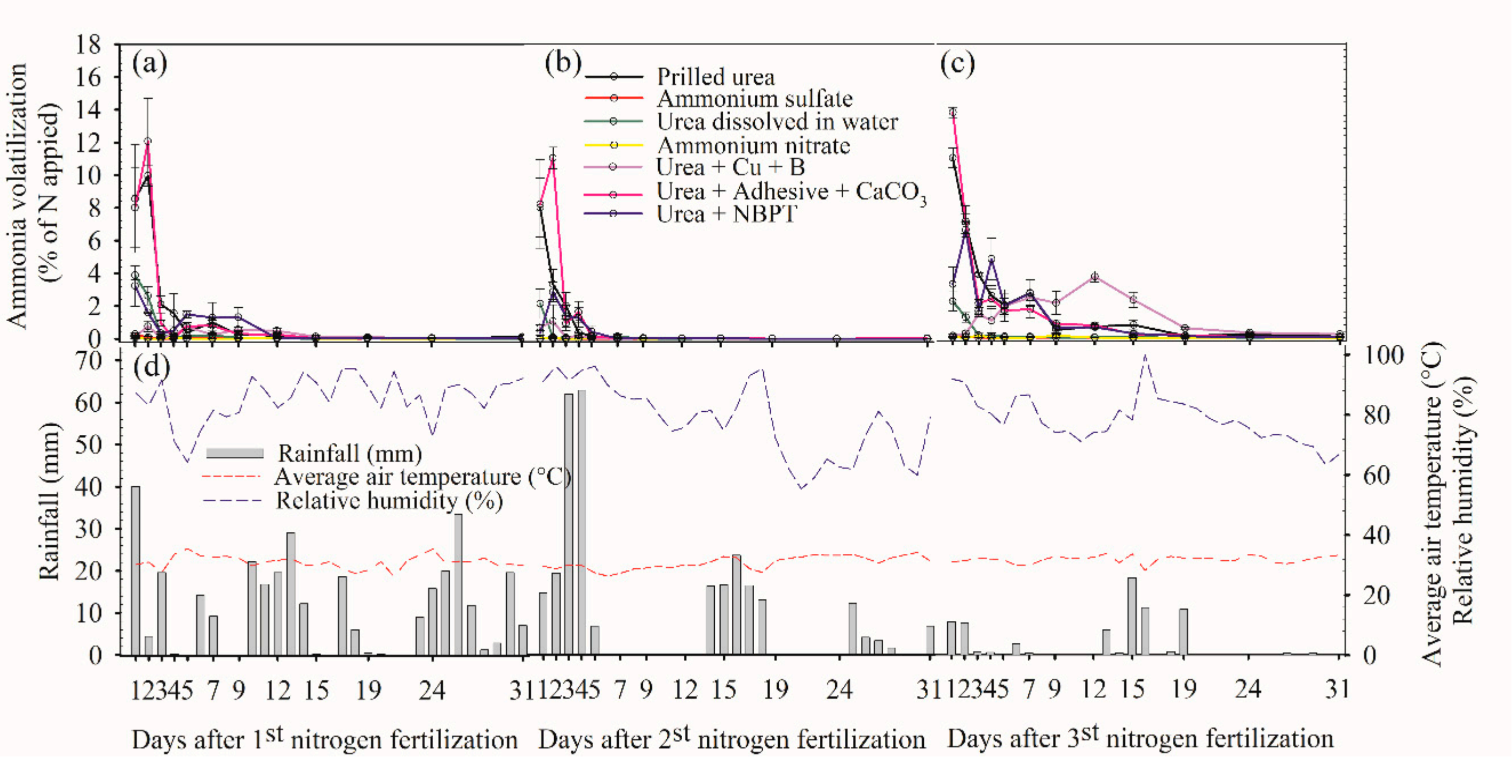

Figure S1

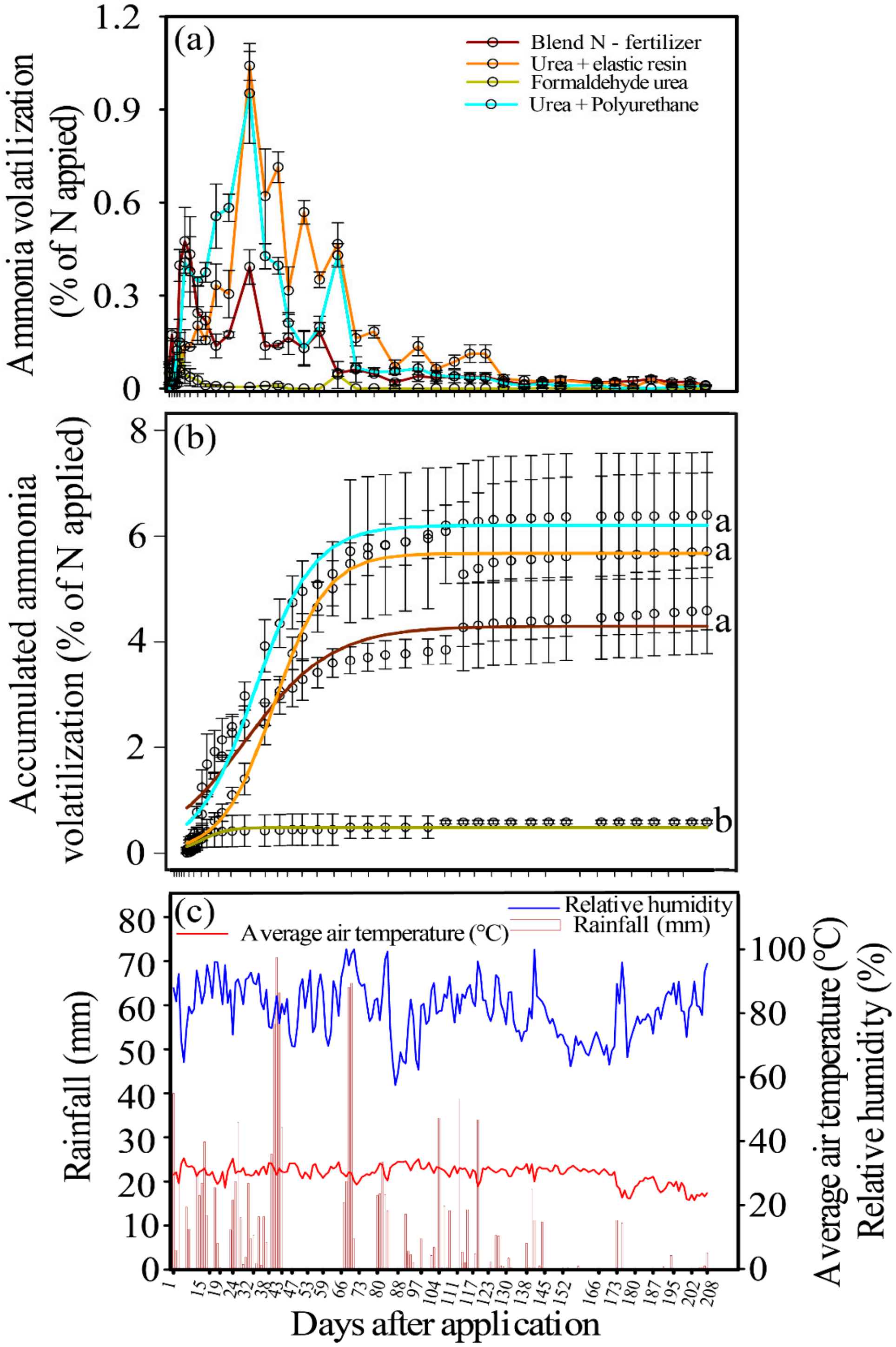

(a)
Blend N - fertilizer
Urea + elastic resin
Formaldehyde urea
Urea + Polyurethane
Ammonia volatilization (% of N appied)
(b)
Accumulated ammonia volatilization (% of N applied)
a
a
a
b
(c)
Relative humidity
Rainfall (mm)
Average air temperature (°C)
Rainfall (mm)
Average air temperature (°C)
Relative humidity (%)
Days after application

Figure S2

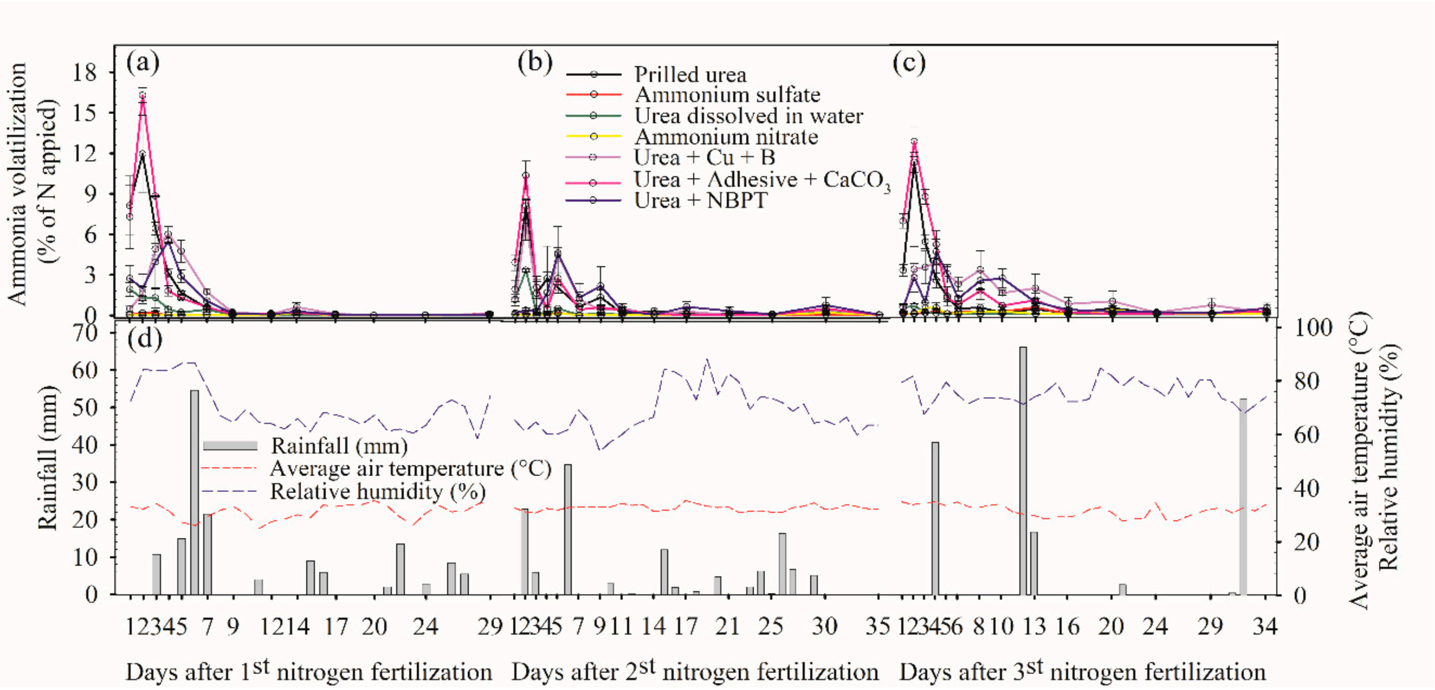

Ammonia volatilization (% of N appied)
(a)
(b)
(c)
(d)
Prilled urea
Ammonium sulfate
Urea dissolved in water
Ammonium nitrate
Urea + Cu + B
Urea + Adhesive + CaCO3
Urea + NBPT
Rainfall (mm)
Average air temperature (°C)
Relative humidity (%)
Average air temperature (°C)
Relative humidity (%)
Days after 1st nitrogen fertilization
Days after 2st nitrogen fertilization
Days after 3st nitrogen fertilization

Figure S3

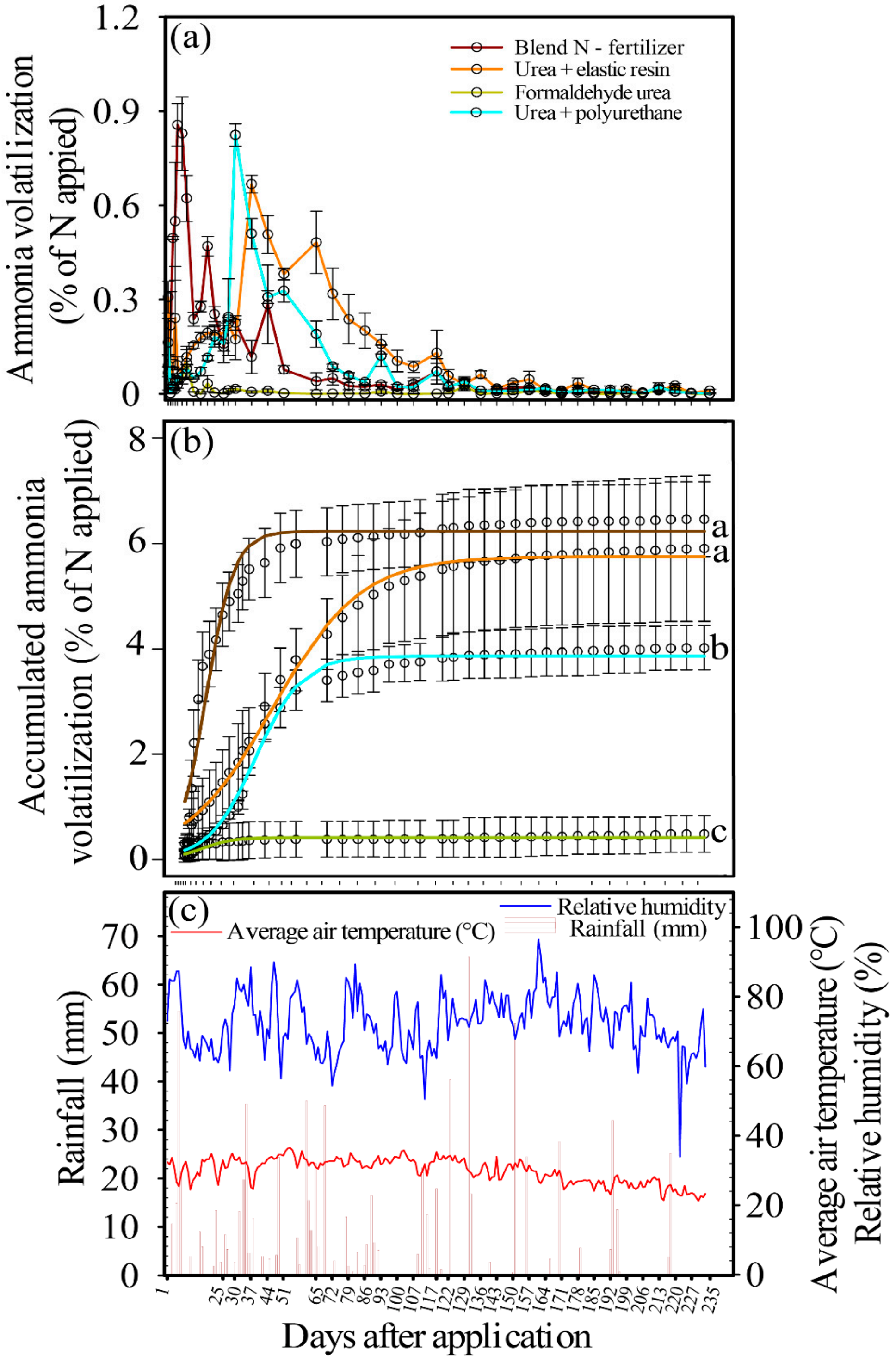
(a)
Blend N - fertilizer
Urea + elastic resin
Formaldehyde urea
Urea + polyurethane
Ammonia volatilization (% of N appied)
(b)
Accumulated ammonia volatilization (% of N applied)
a
a
b
c
(c)
Relative humidity
Average air temperature (°C)
Rainfall (mm)
Rainfall (mm)
Average air temperature (°C) Relative humidity (%)
Days after application

Figure S4

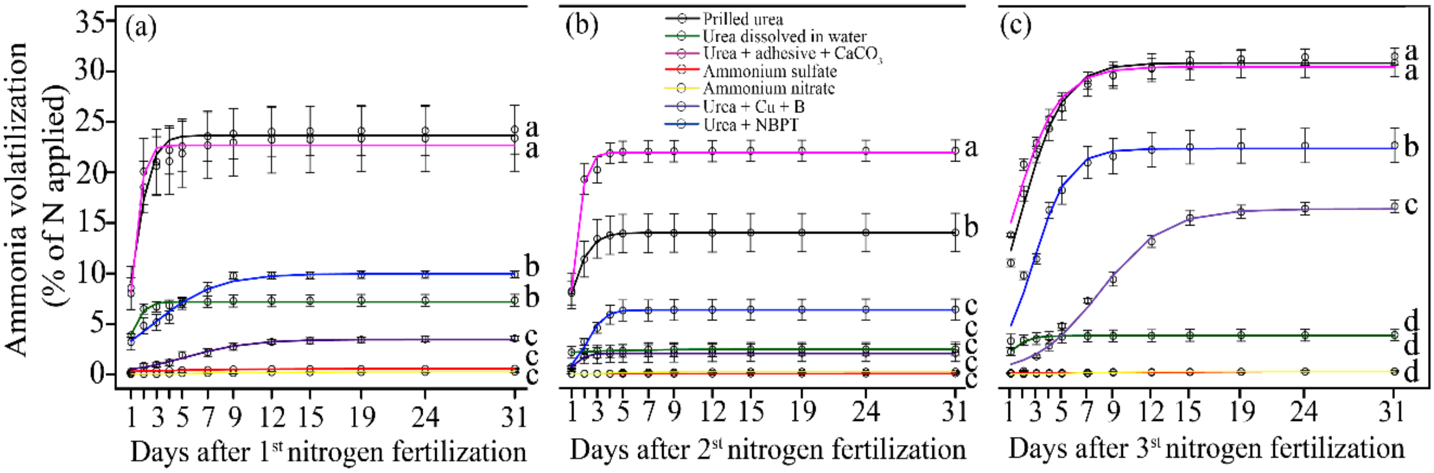

Ammonia volatilization (% of N applied)
(a)
(b)
(c)
Prilled urea
Urea dissolved in water
Urea + adhesive + CaCO3
Ammonium sulfate
Ammonium nitrate
Urea + Cu + B
Urea + NBPT
Days after 1st nitrogen fertilization
Days after 2st nitrogen fertilization
Days after 3st nitrogen fertilization

Figure S5

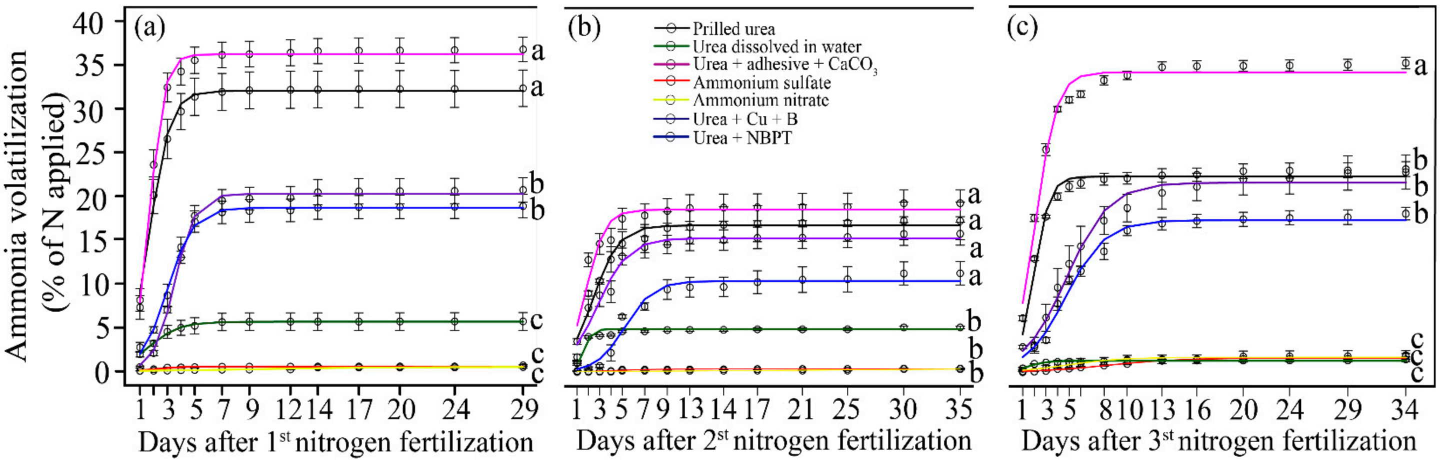

Ammonia volatilization (% of N applied)
(a)
(b)
(c)
Prilled urea
Urea dissolved in water
Urea + adhesive + $CaCO_3$
Ammonium sulfate
Ammonium nitrate
Urea + Cu + B
Urea + NBPT
Days after 1st nitrogen fertilization
Days after 2st nitrogen fertilization
Days after 3st nitrogen fertilization

Figure S6

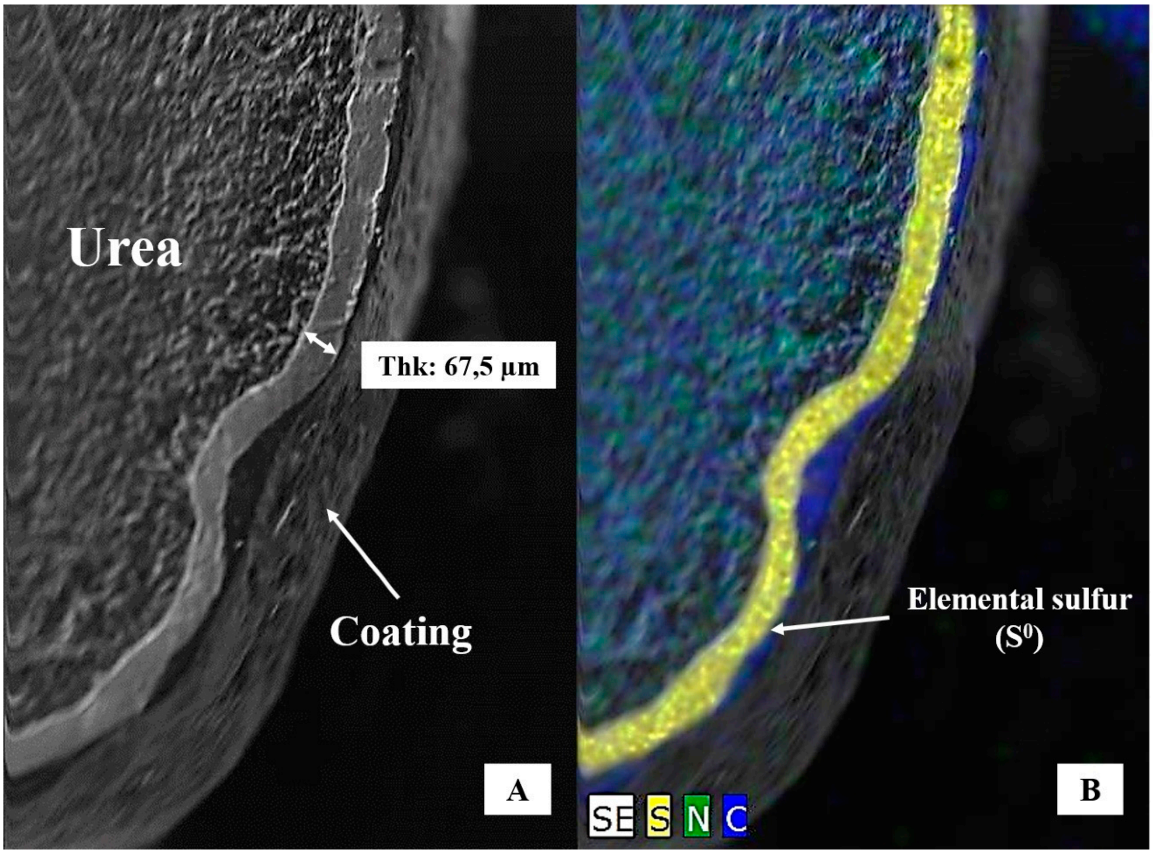

Figure S7

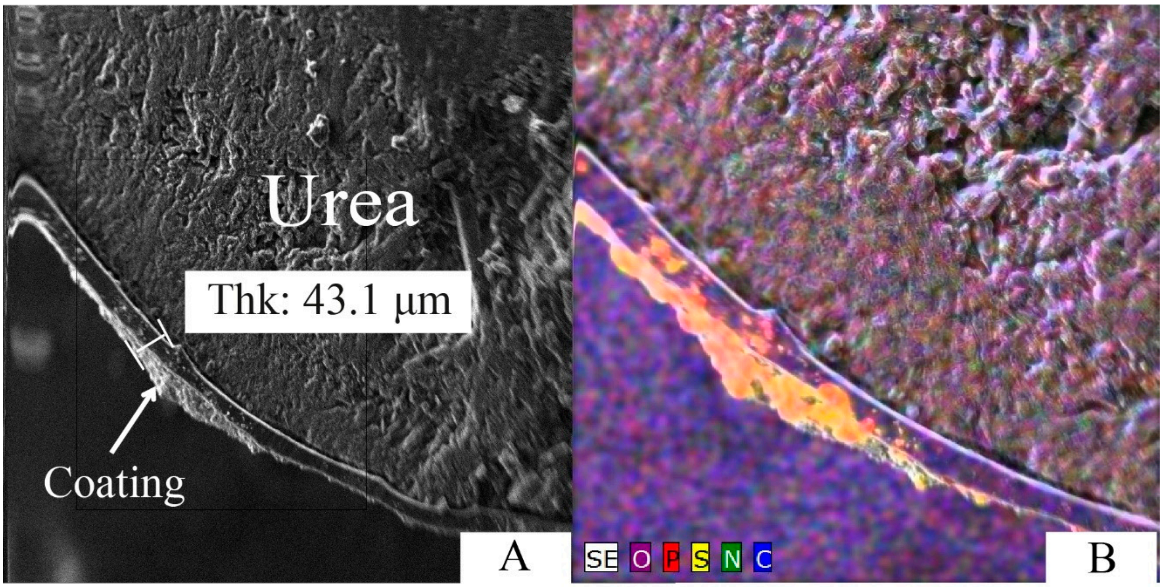

Figure S8

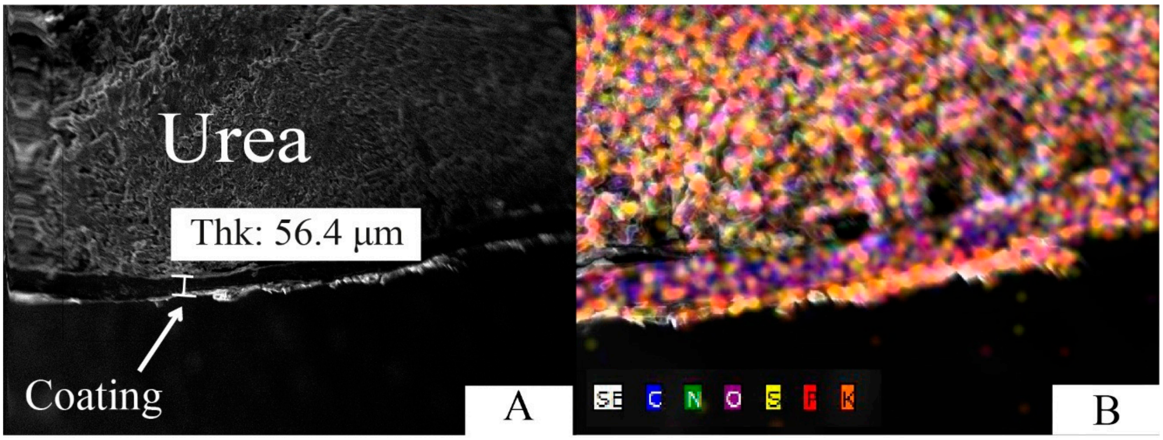

Figure S9

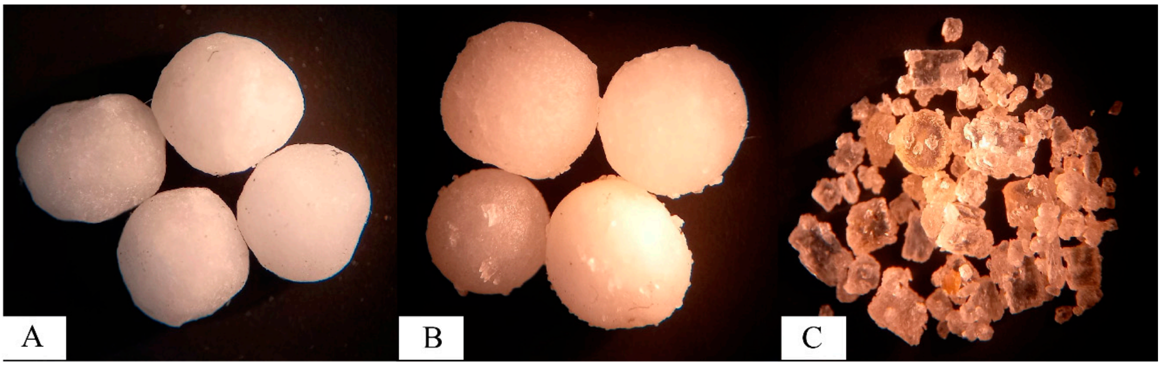

Figure S10

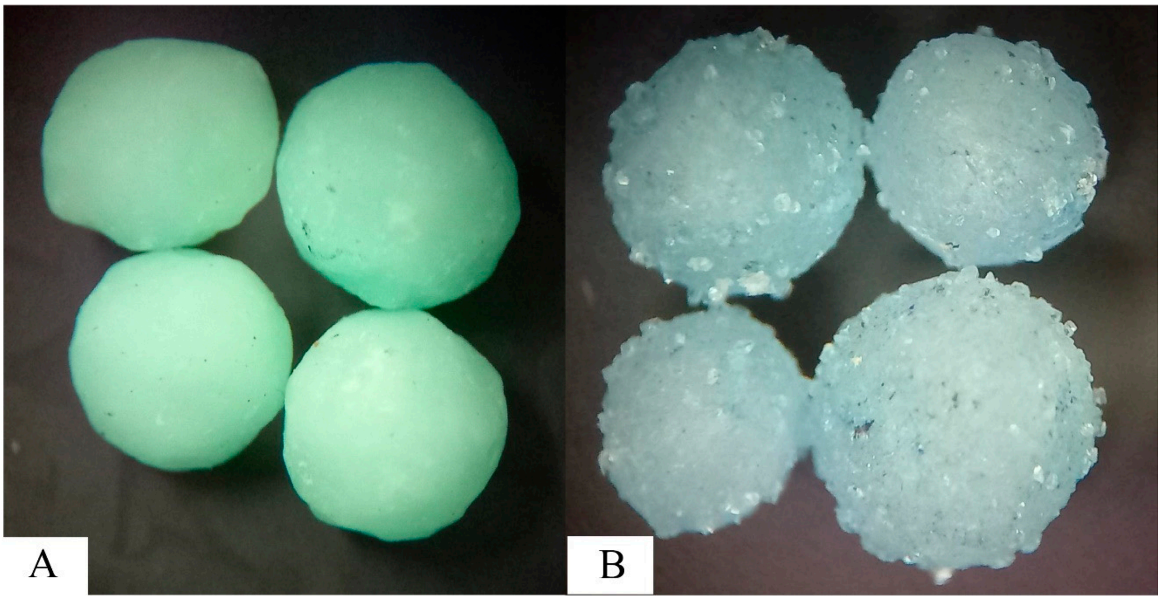

Figure S11

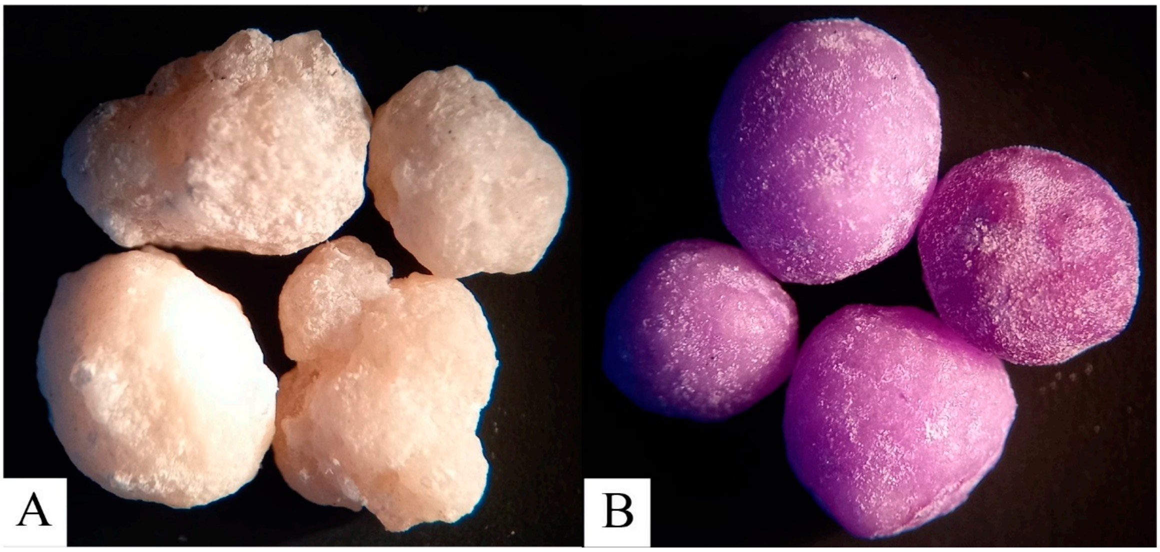

Figure S12

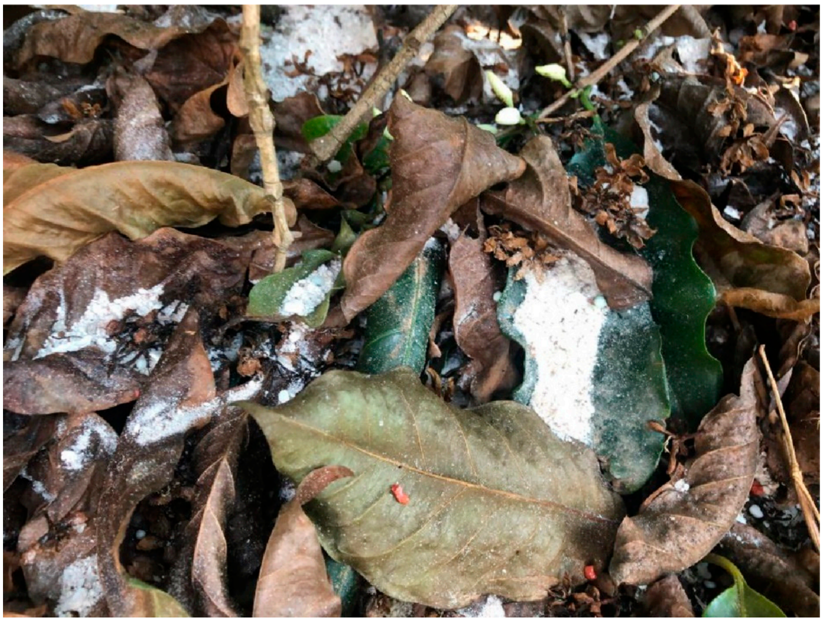

Figure S13
